# Can shared decision-making reduce medical malpractice litigation? A systematic review

**DOI:** 10.1186/s12913-015-0823-2

**Published:** 2015-04-18

**Authors:** Marie-Anne Durand, Benjamin Moulton, Elizabeth Cockle, Mala Mann, Glyn Elwyn

**Affiliations:** The Dartmouth Institute for Health Policy and Clinical Practice, Dartmouth College, Hanover, USA; Department of Psychology, University of Hertfordshire, Hatfield, UK; Informed Medical Decisions Foundation, Boston, USA; Harvard School of Public Health and Boston University Law School, Boston, USA; Boston University Law School, Boston, USA; Support Unit for Research Evidence, Cardiff University, Cardiff, UK; The Dartmouth Center for Health Care Delivery Science, Hanover, USA

**Keywords:** Shared decision-making, Decision-making, Informed consent, Malpractice, Litigation, Decision support techniques

## Abstract

**Background:**

To explore the likely influence and impact of shared decision-making on medical malpractice litigation and patients’ intentions to initiate litigation.

**Methods:**

We included all observational, interventional and qualitative studies published in all languages, which assessed the effect or likely influence of shared decision-making or shared decision-making interventions on medical malpractice litigation or on patients’ intentions to litigate. The following databases were searched from inception until January 2014: CINAHL, Cochrane Register of Controlled Trials, Cochrane Database of Systematic Reviews, EMBASE, HMIC, Lexis library, MEDLINE, NHS Economic Evaluation Database, Open SIGLE, PsycINFO and Web of Knowledge. We also hand searched reference lists of included studies and contacted experts in the field. Downs & Black quality assessment checklist, the Critical Appraisal Skill Programme qualitative tool, and the Critical Appraisal Guidelines for single case study research were used to assess the quality of included studies.

**Results:**

6562 records were screened and 19 articles were retrieved for full-text review. Five studies wee included in the review. Due to the number and heterogeneity of included studies, we conducted a narrative synthesis adapted from the ESRC guidance for narrative synthesis. Four themes emerged. The analysis confirms the absence of empirical data necessary to determine whether or not shared decision-making promoted in the clinical encounter can reduce litigation. Three out of five included studies provide retrospective and simulated data suggesting that ignoring or failing to diagnose patient preferences, particularly when no effort has been made to inform and support understanding of possible harms and benefits, puts clinicians at a higher risk of litigation. Simulated scenarios suggest that documenting the use of decision support interventions in patients’ notes could offer some level of medico-legal protection. Our analysis also indicated that a sizeable proportion of clinicians prefer ordering more tests and procedures, irrespective of patient informed preferences, as protection against litigation.

**Conclusions:**

Given the lack of empirical data, there is insufficient evidence to determine whether or not shared decision-making and the use of decision support interventions can reduce medical malpractice litigation. Further investigation is required.

**Trial registration:**

This review was registered on PROSPERO. Registration number: CRD42012002367.

**Electronic supplementary material:**

The online version of this article (doi:10.1186/s12913-015-0823-2) contains supplementary material, which is available to authorized users.

## Background

While policies are evolving to reflect a progressive shift in medical practice towards patient-centered care [1-4], the approach known as shared decision-making has yet to become incorporated as usual care. King and Moulton have argued that current standards of informed consent are unfit for the rapidly evolving medical landscape [5], where approximately 47% of all medical treatments are “preference-sensitive” [6]. They advocate that adopting shared decision-making would lead to important and necessary reforms in the area of informed consent. In situations of clinical equipoise, also known as preference-sensitive decisions, it is widely argued that individual patient preferences [7] should become the guiding principle for patients making informed decisions together with their healthcare providers [8].

Poor communication and lack of information are the most commonly reported sources of patient dissatisfaction in healthcare [9,10]. Extensive evidence has confirmed that communication failures are strongly correlated with medical malpractice litigation [11-15]. Physicians’ inability to clearly communicate with their patients, to disclose risks and benefits, and to answer their questions, are common predictors of medical malpractice claims [1,16,17]. Levinson analyzed communication behaviors between physicians who had never experienced malpractice litigation and those who had previously been sued, and found that the latter tended to demonstrate poorer communication skills. They were also less likely to form helpful interactions with patients: “relationships matter to both patients and physicians and the relationship itself may be the most powerful antidote to the malpractice crisis that medicine can provide” [11,17].

There is considerable hope that sharing decisions with patients using good communication skills and tools that improve provider-patient communication and understanding of the harm versus benefit trade-offs would lead to lower litigation levels. However, there is yet no evidence to confirm that it is indeed the case. Shared decision-making is defined as involving a patient and health care provider who work together to deliberate about the harms and benefits of two or more reasonable options, in order to choose a course of care that is ideally aligned with the patient’s preferences [18]. Evidence from controlled contexts suggest that shared decision-making can improve patient outcomes by increasing knowledge, realistic expectations, participation in decision-making and reducing post-intervention indecision compared to usual practice [19]. There is uncertainty around its impact on cost and litigation rates [19,20]. Notwithstanding, researchers, policy makers and key stakeholders in this area often speculate that shared decision-making, facilitated by the use of decision support interventions, may reduce litigation rates or limit physicians’ liability in lawsuits [5,21]. In the United States, things are evolving rapidly. Several states (Maine, Vermont, Massachusetts, Minnesota, and Washington) have adopted legislation to promote Shared Decision-Making (SDM) [1]. However there is no widespread adoption of SDM as an alternative to traditional means of obtaining informed consent and no evidence that SDM may lead to reduced litigation.

It is thus important to examine whether SDM (patient participation in decision-making and/or elicitation of patient preferences) and related interventions might reduce preventable litigation. Our aim is to explore the likely impact and influence of shared decision-making and shared decision-making interventions on medical malpractice litigation, and patients’ intentions to initiate litigation.

## Methods

The systematic review protocol was registered on PROSPERO in May 2012 (Registration number CRD42012002367). We planned and reported the review in accordance with the preferred reporting items for systematic reviews and meta-analyses (PRISMA) [22] (see protocol in Additional file 1 and PRISMA checklist in Additional file 2).

### Study selection and inclusion criteria

After removing duplicates and irrelevant studies, three researchers independently screened the title and abstract of retrieved records. Disagreements were resolved by discussion. Two researchers independently screened full-text articles.

We included all observational, interventional and qualitative studies published in all languages, which assessed the effect or likely influence of shared decision-making (patient participation in decision-making and/or elicitation of patient preferences) or shared decision-making interventions on medical malpractice litigation or on patients’ intentions to litigate. Shared decision-making interventions were defined as the use of tools or strategies designed to engage patients in medical decision-making and/or facilitate shared decision-making and patient activation the medical encounter, by providing information about the options and associated outcomes and implicit methods to clarify values [19]. Interventions designed to promote informed consent or communication were included in the review if the standard process of consenting and informing patients was complemented by an effort to involve patients in the decision-making process and elicit their preferences. We included all study outcomes related to litigations.

We excluded studies that exclusively examined the impact of communication skills, provision of information or informed consent on medical malpractice litigation, without considering the influence of patient participation in decision-making and/or elicitation of patient preferences.

### Search methods

The search strategy was developed with an Information Specialist and piloted in OVID MEDLINE (see Additional file 3). We combined keywords and Medical Subject Heading terms for shared decision-making, decision-making, patient participation, doctor-patient relationship, informed decision, decision support, decision support techniques, litigation, medical malpractice, liability, medical negligence claim and legal proceedings (see full list in Additional file 3). The following electronic databases were searched from inception until January 2014: CINAHL, Cochrane Central Register of Controlled Trials, Cochrane Database of Systematic Reviews, EMBASE, HMIC, Lexis Library, MEDLINE, MEDLINE In-Process and Other Non-Indexed Citations, NHSEED, Open SIGLE, PsycINFO and Web of Knowledge. Conference proceedings and the reference list of all primary and review articles were hand searched. A “cited by” search and “related articles” search were also performed on PubMed. We used social media lists to contact 378 individuals registered as having special interests in this area.

### Quality assessment and data extraction

Independent dual data extraction was performed using a piloted pre-designed form. We extracted information about the 1) the author(s)/publication year, 2) type of publication 3) country, 4) source of funding, 5) study purpose, 6) duration, 7) study type, 8) methodological approach, 9) recruitment procedure, 10) theoretical framework, 11) participant characteristics, 12) sample size, 13) setting, 13) type of intervention (if applicable), 14) duration of intervention, 15) follow-up, 16) control condition, 17) methods of analysis, 18) number of participants enrolled, included in analysis, withdrawn and lost to follow-up for both intervention and control groups, 19) outcome measures (type of medical malpractice litigation, outcome of the litigation and factors affecting the outcome, duration of the litigation, litigation cost, accessibility, usability of intervention).

We used Downs & Black quality assessment checklist [23] to assess the quality of observational studies. This checklist was selected for its psychometric properties and relevance for assessing non-randomized studies [23]. Qualitative studies were assessed using a qualitative appraisal tool developed by the Critical Appraisal Skill Programme (CASP)[24]. The quality of case studies was independently assessed by two researchers, using the critical appraisal guidelines for single case study research [25].

### Evidence synthesis

**Figure 1:**
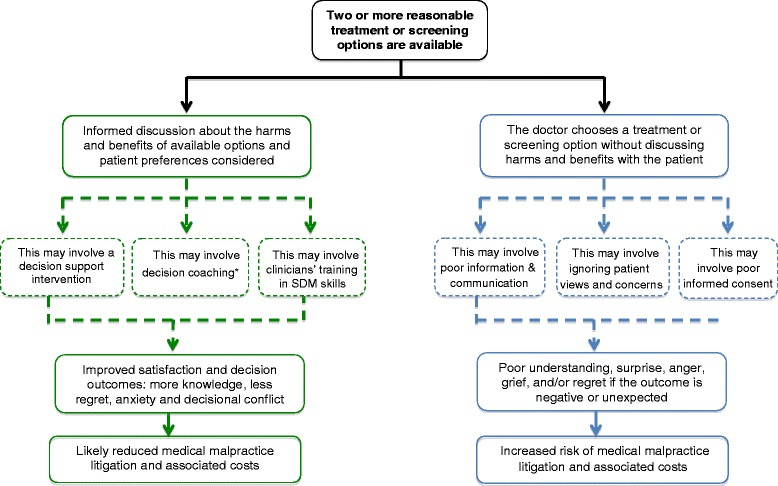
“Theory of change” underlying the narrative synthesis. *Decision coaching involves preparing and facilitating patient participation in medical decision-making in a non-directive manner.

The Economic and Social Research Council (ESRC) guidance for narrative synthesis [26], which we followed and adapted, stipulates that a narrative analysis should be driven by a theoretical model, should include a preliminary synthesis of included study findings, an assessment of the principal trends and relationships in the data, and an examination of the robustness of the findings. For this review, we hypothesized that taking active steps to involve patients in sharing preference-sensitive decisions about their care, such as eliciting individual preferences, would reduce the risk of medical malpractice litigation, actual malpractice suits and related costs (see Figure 1). The ESRC guidance recommends that before undertaking a review, the authors first develop a ‘theory of change’, that describes how the intervention or concept works. The theory of change outlined in Figure 1 was developed prior to data extraction to inform decisions about the review questions and the type of studies to include. A preliminary synthesis was subsequently undertaken using the extracted data organized in a tabular form. The study quality was examined and the relationships and patterns in the data were thematically analyzed and synthesized.

## Results

**Figure 2:**
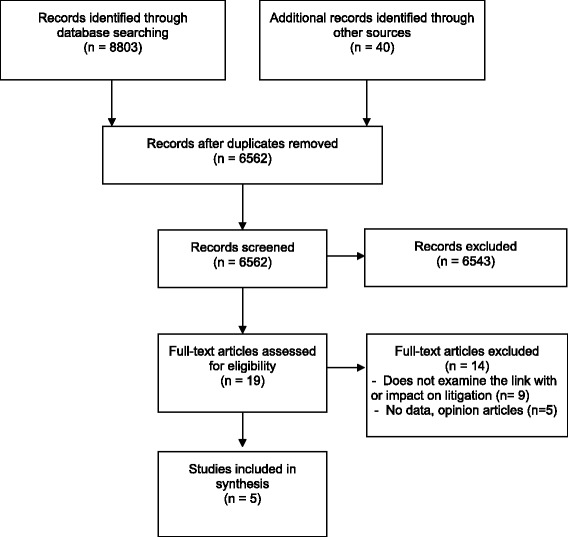
PRISMA flow diagram.

8803 citations were identified from database search and 40 from other sources. After duplicates and irrelevant hits had been removed, 6562 records were screened and 19 articles were retrieved for full-text review. Fourteen studies were excluded upon full-text review for the following reasons: 1) the studies did not evaluate the impact of shared decision-making on litigation or intention to litigate (n = 9); 2) the study did not include any data (e.g. editorial/opinion article) (n = 5). Five articles met our inclusion criteria [27-31] (see Figure 2). We note that 14 editorials and opinion articles, published between 1980 and 2009, hypothesized about the potential for shared decision-making to reduce medical malpractice claims. They were not based on empirical data, and were therefore excluded. A much greater number of citations explored the relationship between poor communication or inadequate informed consent and medical malpractice litigation but failed to consider patient involvement in decision-making, and were thus excluded.

The five included studies report data collected in three countries (United States, United Kingdom and Korea), and were published between 1994 and 2008. They encompass the following study designs: Four qualitative studies (including two case studies), and one quasi-experimental design. The sample size was generally small, ranging from 1 (case studies) to 886 participants. Two studies involved an intervention designed to enhance informed patient choice and shared decision-making: a video-based decision aid for PSA testing [28] and a series of evidence-based leaflets for maternity care [30] (see Table 1). Four studies were conducted with patients from primary and secondary care settings. Subjects taking part in the simulated scenario-based study were recruited from the general population [28]. All participants were adults, mostly white and well educated. None of the studies explicitly reported the use of theoretical models or frameworks.

**Table 1:** **Characteristics of included studies**

**Study**	**Design**	**N**	**Intervention**	**Age**	**Outcomes**	**Conclusion & legal decision**	**Quality scores**
Barry et al. [28]	Quasi-experimental (simulated scenarios)	47	A video-based decision aid for PSA testing	20-70, M = 50	- Focus group voting results: whether the physician met the standard of care.	- Standard of care met if a shared decision-making process is documented in the patients’ notes: voted by 72% of mock jurors	10/26
						- Standard of care met if decision aid used prior to decision-making: voted by 94% of mock jurors	
Beckman et al. [27]	Qualitative study	45	NA	20-80	- Reason for litigation;	- Relationship issues identified in 71% of the depositions.	7/10
					- Specialties of physicians;	- 68% of all issues identified related to the physician’s failure to communicate clearly and transparently and to consider the patient and family views and preferences	
					- Type and frequency of relationship issues;		
					- Who suggested maloccurence.		
Merenstein [29]	Case study	1	NA	53	- Causes and outcome of the medical malpractice trial	Dr Merenstein’s residency was found liable for not meeting the standard of care, despite having complied with the principles of shared decision-making, evidence-based medicine and the National guidelines.	6/29
Stapleton et al. [30]	Qualitative study (observation and in depth interviews)	886	Evidence-based leaflets for pregnancy	Not known	- Participants’ views on the use of evidence-based leaflets and its influence on litigation	Health care providers felt that ordering more tests and procedures offered better protection against litigation than promoting evidence-based leaflets and patient preferences.	7/10
Um [31]	Case study	1	NA	33	- Causes and outcome of the medical malpractice trial	An obstetrician who discounted his patient’s wish to undergo amniocentesis testing was found guilty for interfering with the patient’s self-determination, after the plaintiff gave birth to a baby diagnosed with Down’s syndrome.	14/29

### Robustness of the synthesis

The qualitative studies (n = 2, all designs except case studies) that were rated against CASP had satisfactory quality ratings (see Table 1) [27,30]. For both studies, the research design, sampling and data collection procedures were deemed appropriate. Reflexivity, ethical issues and analysis could have been improved. The quality of the case studies (n = 2) was low; these were exclusively based on documented legal cases [29,31]. Areas of concerns for both case studies where the absence of dual analysis involving an independent researcher or triangulation, the lack of clearly formulated questions and conceptual framework and lack of information about the data collection and data analysis procedures. Findings should therefore be interpreted with caution. The quality of the quasi-experimental study was low, but consistent with Downs and Black’s average ratings for non-randomized studies [28]. Given this study was based on a simulated court case, where lay people were asked to behave as hypothetical jurors, external validity was poor and internal validity was low with a high risk of selection bias and other confounders.

### Narrative synthesis

The relationships and patterns occurring across the five included studies were thematically analyzed and synthesized. Given the heterogeneity of the included outcomes, it was not possible to synthesize the data according to their outcome measures (se Table 1). The following themes, closely linked to the study outcomes, emerged: 1) Interfering with patient preferences; 2) Documenting shared decision-making to meet the standard of care; 3) Can a decision aid offer medico-legal protection? 4) Using “defensive medicine” to minimize malpractice litigation Three out of four themes are closely aligned with the theory of change, which we had developed before undertaking this analysis and which is represented in Figure 1. However, theme 4 is one that naturally emerged from the data analysis, and which we had not anticipated or identified as prominent.

#### Theme1: Respecting patient preferences

One might assume that medical malpractice claims are primarily triggered by an unexpected significant adverse outcome, such as death. Beckman’s descriptive case review [27] and Um’s case study [31] suggest that other factors, such as the quality of the doctor-patient relationship, the consideration and respect for patient preferences, poor communication and patients’ involvement (or lack of involvement) in the care and decision-making processes, influence, or may even determine, the initiation of malpractice claims. In a qualitative analysis of 45 plaintiff depositions of settled cases, Beckman et al. extracted information about the reason(s) motivating the claim, all information pertaining to the relationship between the claimant and health provider, and whether a health professional suggested maloccurrence (i.e. a negative outcome that is not imputable to the quality of care provided by the medical team). The authors independently coded the verbatim transcripts, and identified 15 issues and their respective frequencies. Their analysis suggested that problematic patient-provider relationship issues had occurred in 71% of all depositions (inter-rater reliability of 93.3%). The following four categories emerged: not understanding the patient and/or family perspective (13.1%), dysfunctional delivery of information (26.4%), devaluing patient and/or family views (28.9%), and patient abandonment (31.6%). Three of these categories (not understanding the patient and or family perspective, devaluing patient and/or family views and patient abandonment) are likely to be associated with the physician’s inability to promote and support the patient and family’s involvement in shared decision-making, and consider their concerns and preferences.

Recurrent in Beckman’s analysis was the clinicians’ tendency to ignore patients’ views, thus infringing on their autonomy and interfering with their preferences. Examples of specific issues relating to shared decision-making included: failure to solicit patient and/or family opinion (2.6%), discounting a patient and/or family opinion (5.3%), discounting a family’s attempt to advocate (5.3%), not listening (5.3%), failure to keep a patient and/or family up to date (5.3%).

Um’s case study of a pregnant woman’s struggle to receive care and procedures that are aligned with her preferences [31] illustrates the same theme. The lack of maternal involvement in deciding about prenatal testing led her to sue her physician after her newborn baby was diagnosed with Down’s syndrome. Over the course of the pregnancy, the pregnant mother, who was aware of family history of chromosomal abnormality, had repeatedly requested an amniocentesis. Despite her concerns, and repeated requests for further invasive testing, the clinician refused to arrange the procedure. The obstetrician was sued on the grounds of negligence and found liable for interfering with the “mother’s right to self-determination”, thus interfering with her preferences [31]. Her views and preferences had clearly been expressed, but were overruled by the health care provider. In this particular instance, a lawsuit could have been avoided if the provider had respected her explicit and informed preferences.

Using data from actual malpractice lawsuits in different contexts and clinical areas, both studies demonstrate that over and above the importance of good communication, the inability to involve patients in decision-making and to consider their concerns and preferences can incite patients to commence litigation. In Beckman’s study, the authors insist on the impact of “devaluing a patient’s or family views” on medical malpractice intentions, referring to “particularly risk-laden form of sharing information”.

While the above analysis indicates that discounting patients’ views might increase the risk of litigation, one of five studies included in this review examined the implications of discussing the pros and cons of PSA testing [29]. In 1999, when advising a 53 year old patient, Dr Merenstein, reports testifying that he followed the principles of shared decision-making as well as the US National guidelines for prostate cancer screening (Guidelines of the American Academy of Family Physicians, the American Urological Association and the American Cancer Society). He discussed the pros and cons of screening for a disease that, if left undiagnosed, may not be life threatening, and described the poor accuracy and potential harms of the PSA test. The patient subsequently declined to have PSA screening. The discussion and the patient’s decision were documented by Merenstein. A few years later, the patient saw another Physician who ordered a PSA test without discussion. This test led to a diagnosis of prostate cancer. The plaintiff denied that an informed discussion about the risks and benefits of the PSA test had occurred. There was testimony from two physicians, on behalf of the plaintiff, that the standard of care in Virginia was to not discuss the uncertainties of testing, but rather to perform the test. Dr Merenstein’s residency program was found negligent because of the previous failure to perform a PSA test. Dr Merenstein and a defense expert testified that Merenstein had nevertheless followed published clinical practice guidelines, had discussed the harms and uncertainties of the test, and promoted shared decision-making and his patient’s autonomy. The jury and the plaintiff’s lawyers did not recognize this process as being consistent with the standard of care. While Dr Merenstein was not found negligent, his residency program was found liable and the case was settled without further appellate review. It is important to recognize that there are many variables that may have influenced the jury’s decision.

#### Theme 2: Documenting shared decision-making to meet the standard of care

In response to the Merenstein trial, Barry et al. conducted a simulation study [28] to investigate whether involving patients in PSA screening decisions would influence a jury’s verdict, and examine whether the Merenstein outcome had been atypical. Lay participants were divided into six focus groups and instructed to behave as hypothetical jurors in considering two variations of the Merenstein case. In the first variation, narrated to the first three groups, the physician’s notes did not mention a discussion about the risks and benefits of the PSA screening (“no pros and cons note” scenario). In the second variation, presented to the remaining three groups, the physician had clearly documented a discussion about the harms and benefits of PSA screening, resulting in the patient’s informed decision to decline the test (“pros and cons note” scenario). All potential jurors were asked to decide whether the physician had met the standard of care, and if not, whether any harm had been caused. The majority of participants (83%) in the first three groups considered that there was deviation from the standard of care, i.e. negligence, and no informed consent. As one participant commented: “*Not documented, not done.”*

Further, 61% of the participants in the first three groups also believed that harm had been caused. Although there is no clear evidence that ordering the PSA test would have affected the cancer prognosis, the majority of mock jurors in the first three groups (61%) believed that ordering the test would have saved the patient’s life or significantly improved the outcome. In their words: *“Because of the severity of the disease, the doctor should have done the test as a standard process. Even if he explained the pros and cons, I don’t think there should be a question of him not doing the test. He should do it as a standard process with no discussion.”* This reflects a trend to use tests and invasive procedures, even if the benefits are unclear and some individuals might thus prefer to decline the test when informed. The participants’ view was reminiscent of the Merenstein judgment.

**Figure 3:**
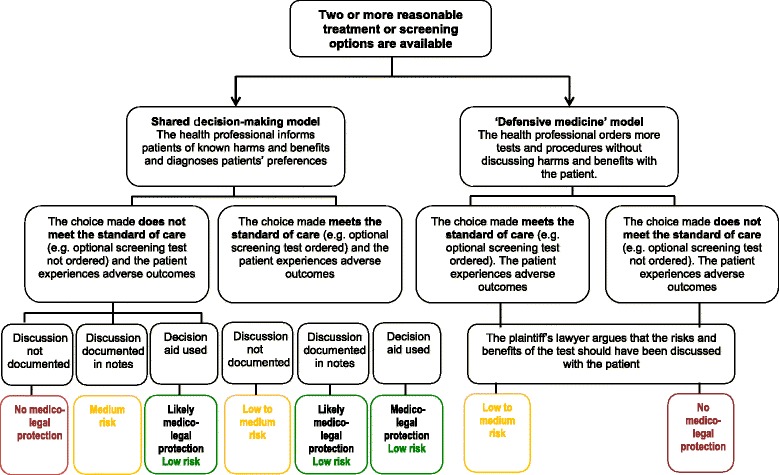
Liability risk according to patient involvement in decision-making.

However, when presented with the “pros and cons scenario”, 72% of all participants considered that promoting patient choice and facilitating shared decision-making met the standard of care, provided the discussion had been documented in the patient’s record. Contrary to the outcome of the Merenstein trial, these findings indicate that embedding and documenting shared decision-making in routine clinical practice could provide a higher degree of medico-legal protection and lead to better informed consent. However, this should be interpreted in light of contextual factors and study limitations. First, the vote for the second scenario (pros and cons) was not unanimous: 28% believed that the standard of care had not been met, and 23% felt that harm had been caused. Although a minority vote, it mirrors the outcome of the Merenstein case. Second, given the simulated nature of the study, it is difficult to infer whether this outcome would be representative of similar malpractice results. However, these findings indicate that shared decision-making documented in the patient’s record could provide what King & Moulton call ‘perfected informed consent’ and, in many instances, may prevent litigation on a failure to inform claim (see Figure 3).

#### Theme 3: Can a decision aid offer medico-legal protection?

As part of Barry‘s study, and after the initial set of votes, all participants were shown a video-based patient decision aid for PSA screening. They were asked to imagine that the decision aid had been provided to the patient prior to his informed refusal, and documented in the notes. The vote was almost unanimous (94%), confirming that the standard of care would have been met without any harm caused. One participant said: “*The tape tells you as much as you can possibly tell a patient.”* And another commented: “*Let me tell you something, after watching that video, there’s no way that you could not know what it is, the pros and cons, the risks, the quantity, the quality of life, the incontinence, impotence…. Honestly that’s even better than the doctor just saying it to you.”* After being shown the decision support intervention, the majority of participants implicitly conceded that offering a choice and discussing the pros and cons were justified, and eventually gained understanding of the harms, benefits and controversies of the test. Although this finding is based on a simulation study, it suggests that documenting the use of decision support interventions could be considered the highest standard of care, and could reduce the risk of liability.

#### Theme 4: Using “defensive medicine” to minimize malpractice litigation

Two out of the five included studies [29,30] highlight that a number of health professionals believe that promoting the use of technological interventions offers the best protection against litigation. In a maternity clinic, Stapleton and colleagues observed and interviewed clinicians about their use, opinion and perceived potential impact on litigation of evidence-based information leaflets. According to their observations, obstetricians showed a tendency to minimize, or not mention, the risk of interventions, treatments or screening procedures. Some obstetricians explicitly favored ordering tests and treatments and were not curious to know the views of their patients. They strongly believed that their own clinical recommendation offered better medico-legal protection than adopting an approach based on informing patients and adopting a shared decision-making approach. This view was not shared by the midwives who, by and large, promoted the use of the decision support interventions. Patients in this study reported feeling ‘bullied’ into undergoing tests and procedures.

## Discussion

The assertion that involving patients in decision-making leads to less litigation has not been extensively studied and cannot yet be confirmed. No empirical research conducted in clinical settings has assessed the impact of shared decision-making and related interventions on medical malpractice litigation. We are thus unable to determine whether or not shared decision-making and related interventions can reduce malpractice litigation. However, three out the five studies analyzed here provide retrospective and simulated data suggesting that not paying attention to patient preferences, particularly when no effort has been made to inform and support understanding of possible harms and benefits, may put clinicians at a higher risk of litigation. However, given the number, heterogeneity and quality of included studies, these findings should be interpreted with caution. Simulated malpractice scenarios indicate that supporting and documenting shared decision-making in the patients’ notes, as well as using decision support interventions to support patients’ deliberation, would meet the standard of care, and as such, could offer ‘perfected informed consent’ and might reduce litigation. Nonetheless, two studies also emphasize that health professionals are still wary of promoting and respecting patient preferences. Many continue to believe that ordering more tests and procedures, irrespective of patient informed preferences, is a better defense against litigation than the promotion of patient’s autonomy and informed preferences. This is a complex issue, and one that might discourage clinicians from practicing SDM. Further, it is worth noting that adopting shared decision-making without accurately documenting the process, even when decisions are aligned with the recommendations in clinical guidelines, would not be recognized from a medico-legal perspective as dispositive of the issue (see Figure 3).

### Strength and weaknesses of the study

Several limitations need to be considered. First, studies that met our inclusion criteria were rare and highly heterogeneous, two of which were court-based case studies in different countries. We decided not to restrict the type of studies included as we were aware of the lack of data in this area and wanted to have as comprehensive a perspective as possible. Synthesizing results of such diversity can be problematic and unreliable. In order to limit interpretation biases, we closely followed the guidelines provided by the ESRC framework. We also considered the impact of contextual factors, particularly for the included case studies. For instance, the Merenstein verdict [29] was in opposition with the findings of Beckman’s and Um’s studies [27,31]. These apparently contradictory rulings were cautiously interpreted in light of contextual factors. The Merenstein case study is a self-reported case study published in the Journal of the American Medical Association. Its scientific quality and validity are therefore limited, as demonstrated by the quality assessment, but met the inclusion criteria and provided a unique account of the possible consequences of promoting shared decision-making in naturalistic settings. We do not know whether the Merenstein case is an outlier or whether its outcome may have been typical or representative of similar malpractice litigation cases. However, it is important to bear in mind that despite the impact that the case and self-reported commentary have had over the past eight years, it has absolutely no precedential value as case law. Finally, and in relation to Barry’s study, it is worth noting that, in medical malpractice, the determination of whether or not a ‘standard of care’ is met is typically determined by medical experts, and not by lay people, although the jury would make the final decision. The findings of Barry’s study thus need to be interpreted carefully. In a real case of medical malpractice, the jury’s final decision might have been influenced by the experts’ discussions and opinions as to whether or not the standard of care had been met, which was not the case in Barry’s study.

### Comparison with other studies

Ample evidence suggests that patients are less likely to sue their physician when the relationship is satisfactory and the provider displays person-centered communication skills [17,32-34]. However, as demonstrated by this review, no studies have empirically examined the effect of shared decision-making on lawsuits. This may be due to methodological challenges. Litigation is a relatively rare occurrence. A very large sample size and long-term follow-up would be required to evaluate, in a controlled setting, the exact impact of shared decision-making on litigation incidence and costs. Nonetheless, for over two decades, many have advocated for shared decision-making’s role in informed consent procedures [21,35-38]. Green, in 1988, urged physicians and patients to engage in collaborations and agreements about the optimal course of care, to document it in the patient’s chart, and use “questionnaires that elicit values, preferences and needs” which we now refer to as decision support interventions [35].

More recently, King and Moulton examined the principles underpinning informed consent law in the United States and exposed that existing medical consent procedures (i.e. the physician and patient based standards) are not aligned with advances in medicine, easy access to information, growing expectations, and recent policy developments promoting patient autonomy and involvement in health care [5]. The authors suggest a revision of informed consent doctrine, and propose shared decision-making as a prerequisite and adjunct to the informed consent process between provider and patient for preference-sensitive care or situations of clinical equipoise.

This proposition is supported by empirical evidence on the effectiveness of informed consent procedures [38-41], suggesting that informed consent is practiced today through the “ritualistic recitation of risks and benefits” [42], enumerated on a written form with little or no engagement of the patient and little or no evaluation of whether the patient understands any of the risks. There is no bidirectional discourse. Most clinicians feel that informed consent is the patient signature on a piece of paper and do not recognize that informed consent is the process of communication between provider and patient and that risks, benefits and alternatives are an essential part of that discussion. In a content analysis of hospital informed consent forms, Bottrell et al. [38] established that risks were not systematically portrayed and the majority of consent forms were used as mere treatment authorizations, regardless of the quality of the consent process undergone. Patients cannot therefore appreciate risks and make an informed decision if the harms, benefits and alternative options are not presented in a comprehensive and accessible manner and may later regret having consented to treatments they had very little knowledge of. The shortcomings of current consent procedures could be remedied by promoting shared decision-making in the clinical encounter and the use of decision support interventions, thus ensuring that patients are thoroughly aware of all aspects of the treatment or screening options available before engaging in a course of care [38,43-46]. Promoting shared decision-making and decision support interventions would contribute to ensure that patients’ preferences and self-determination are respected, and patients are fully informed of all possible outcomes, harms, benefits and alternative health options. If such process had occurred in the context of Um’s case study, the lawsuit would have been avoided and the patient would have received the desired course of care.

However, a sizeable proportion of health professionals continue to practice defensive medicine and believe in the virtue of ordering more tests and procedures to avoid or reduce litigation risks [47-49]. Gattelari at al. confirmed people’s common tendency to opt for optional tests when they possess poor knowledge of the harms and benefits of available options [50]. When presented with hypothetical scenarios and no explanations of the harms and benefits of PSA screening or mention of National Guidelines, the majority of participants felt that the test should have been ordered, and the GP found liable for the patient’s negative outcomes. Further, a study of the impact of National guidelines on physicians’ perceptions of medico-legal risks for PSA screening found that 46% of surveyed GPs still perceived medico-legal risk if the test was not performed, despite clear National guidelines [47].

## Conclusions

The desirability of patient involvement in medical decision-making and its role in informed consent procedures have been advocated for decades. Many have assumed that promoting shared decision-making would reduce preventable litigation. However, there is to date no clear evidence to confirm that shared decision-making can indeed reduce medical malpractice litigation. Given the number, heterogeneity and quality of included studies, the review provides insufficient evidence to draw firm conclusions. Data from retrospective and simulated studies seem to indicate that shared decision-making, and the use of good quality decision support interventions, could offer some level of medico-legal protection. It also highlights some clinicians’ reticence to consider that a patient who has understood the risks and implications of various tests, treatments or surgical procedures, might be less likely to sue. Many clinicians believe that their own medical opinion, combined with more tests and procedures, remains the best protection against litigation, as illustrated by the jurors’ verdict in the Merenstein judgment.

While most countries are yet to embed shared decision-making in legal reforms of informed consent, Washington State passed legislation in 2007 to change the informed consent law and offer physicians who practice shared decision-making with a “certified “ decision aid, a higher degree of protection against a failure to inform law suit. There are now five states that have promoted shared decision-making in state law. Massachusetts now requires that in order to be a medical home or an accountable care organization, the state must certify the entity, and it must, in turn, encourage shared decision-making for certain preferences sensitive conditions in order to qualify.

Shared decision-making, and the use of decision support interventions, will only offer an effective alternative to current informed consent procedures if it becomes embedded in legislative health policy reforms and part of common law. It constitutes an overwhelming ethical imperative in the context of preference sensitive care where the failure to elicit and act on patients’ preferences is tantamount to operating on the wrong patient [51]. Nevertheless, more empirical data is needed to determine the impact of shared decision-making on preventable litigation and potentially overcome clinicians’ reticence and the illusory protection that defensive medicine seems to provide.

## Additional files

Additional file 1:
**Systematic review protocol.**
Additional file 2:
**PRISMA checklist.**
Additional file 3:
**Search strategy.**

## Abbreviations

ESRC: Economic and social research council
PSA: Prostate specific antigen (test)
SDM: Shared decision-making
