# Supplementary material for: Can shared decision-making reduce medical malpractice litigation? A systematic review
Source: BMC Health Serv Res. 2015 Apr 18;15:167. doi: 10.1186/s12913-015-0823-2 (PMC4409730; doi:10.1186/s12913-015-0823-2)
Supplement: Additional file 1: — Systematic review protocol. [file 12913_2015_823_MOESM1_ESM.docx]

**Systematic Review Protocol**

**Can Shared Decision-making Influence Medical Malpractice Litigation?**

Durand M.A., Moulton B., Mann M., Elwyn G.

# 1. Background

While policies in the UK are progressively evolving to reflect current trends and changes in medical practice, such as growing patient autonomy and access to information (Bal and Choma 2012), Shared Decision-making is yet to influence changes in legislative frameworks. To date, the practice of medicine, health services research and innovations, have generally failed to influence our legal standards. King and Moulton (2006) argued that current standards of informed consent are unfit for the rapidly evolving medical landscape, where approximately 47% of all medical treatments are “preference-sensitive” (Godlee 2005), therefore warranting shared decision-making, and necessary reforms of informed consent. In situations of clinical equipoise, also known as preference-sensitive decisions, patient preferences and attitudes to risks vary between individuals and circumstances (Feldman Stewart 2000), and are crucial in determining their preferred course of care. Current legal standards of informed consent are based on the needs of a “reasonable patient”, which, in the context of Shared Decision-making may limit the amount of information, involvement and personalised care required by patients to make a high quality informed decision (King and Moulton 2006). Further, it is worth noting that poor communication and lack of information are the most commonly reported sources of patient dissatisfaction (Coulter et al. 2001; Grol et al. 2000).

Shared Decision-making is a process in which patients are involved as active partners with their clinicians to clarify acceptable options and choose their preferred course of care, one that is ideally aligned with their values and preferences. There is documented evidence that Shared Decision-making can improve outcomes for patients, clinicians, commissioners and the population by increasing knowledge, realistic expectations, participation in decision-making and reducing decisional conflict and post-intervention indecision compared to usual practice (Stacey et al. 2011). Other likely benefits of Shared Decision-making include reductions in the uptake of elective surgical procedures, reduction of unwarranted practice variation and reduced litigation costs. The evidence behind the cost reduction and impact on litigations is scarce. In the latest Cochrane review of decision aids, none of the 86 studies included for review examined the impact of Shared Decision-making on litigations (Stacey et al. 2011).

Researchers, policy makers and key stakeholders in this area often rely on the widely accepted assumption that shared decision-making can reduce medical malpractice litigations and associated costs (King & Moulton 2006). However, at present, there is no empirical evidence confirming this assumption. While the link between poor information and patient dissatisfaction has been clearly established, little is known about the impact of shared decision-making and its key components; effective communication, truly informed consent, patient involvement and elicitation of patients’ values and preferences, on medical litigations. Further evaluation is required.

This current lack of empirical evidence is partly justified by less than two decades of research in this field and obvious methodological constraints. A very large sample size and long-term follow-up would be required to evaluate, in a trial, the exact impact of Shared Decision-making on litigation costs. However, observational and qualitative data of the likely impact of shared decision-making on litigations exist, but has not yet been examined in a systematic review.

# 2. Aims and Objectives

Our aim is to explore the impact of shared decision-making (and key components: patient participation in the decision-making process and elicitation of values and preferences) on medical malpractice litigation.

Our objectives will be:

- To investigate the impact of shared decision-making interventions on medical malpractice litigation;

- To assess whether effective doctor-patient communication, truly informed consent, patient involvement in medical decision-making and the elicitation of patients’ values and preferences can influence healthcare litigations;

- To infer the impact of shared decision-making on litigation costs.

# 3. Research questions

- What is the link between doctor-patient communication, informed consent, patient involvement and medical malpractice litigation?

- Can better communication in healthcare, truly informed consent and patient involvement in medical decision-making reduce malpractice litigation?

- Can shared decision-making influence litigation costs?

# 4. Study selection criteria

## 4.1 Types of studies

We will include all observational, interventional as well as qualitative studies published in all languages that:

1. **Assess the effect of** shared decision-making interventions on medical malpractice litigation

2. **Asses the effect of interventions designed to improve** doctor-patient communication and/or truly informed consent and/or patient involvement in medical decision-making and/or elicit patient values and preferences on medical malpractice litigation

3. **Explore the influence of shared decision-making** and/or key components of shared decision-making (truly informed consent and/or patient involvement in medical decision-making and/or elicit patient values and preferences) on medical malpractice litigation or intention to litigate.

We will exclude studies that only examine communication skills, provision of information or informed consent without considering the importance of patient participation in decision-making and elicitation of preferences.

## 4.2 Types of interventions

We will include all interventions designed to engage patients in medical decision-making and/or facilitate shared decision-making and patient involvement or activation. We will also include all interventions designed to improve doctor/patient communication and/or improve informed consent and/or improve patient involvement in medical decision-making and/or elicit patient values and preferences. Interventions designed to promote informed consent will only be included if the standard process of consenting and informing patients about the options available is complemented by an effort to involve patients in a discussion with their health professional about all possible treatments, procedures and associated risks and benefits. For the purpose of this review, this concept will be defined as truly informed consent. All formats of interventions will be included: web-based, video, audio and printed materials.

Studies that explicitly assess the influence of shared decision-making and/or doctor/patient communication and/or truly informed consent and/or patient involvement in medical decision-making and/or the elicitation of patient values and preferences on medical malpractice litigation will be included even if they do not involve an intervention.

## 4.3 Types of outcome measures

We will include all outcome measures, but are particularly interested in the following primary and secondary outcome measures.

**Primary outcome measures**

- Type of medical malpractice litigation;
- Outcome of the litigation and factors affecting the outcome;
- Duration of the litigation.

**Secondary outcome measures**

- Litigation costs

# 5. Search strategy

We will perform a comprehensive literature search to identify relevant studies in the following electronic databases (see appendix 1).

| CINAHL (*Cumulative Index to Nursing and Allied Health Literature)* | 1982 – 2012 |
| --- | --- |
| Cochrane Central Register of Controlled Trials | 1996 – 2012 |
| Cochrane Database of Systematic Reviews (CDSR), | 1996-2012 |
| EMBASE | 1980 -2012 |
| HMIC (*Health Management Information Centre*) | 1979-2012 |
| Lexis Library | 1980-2012 |
| MEDLINE | 1946 – 2012 |
| MEDLINE In-Process and Other Non-Indexed Citations | 1951-2012 |
| NHSEED (*NHS Economic Evaluation Database*) | 1979 – 2012 |
| Open SIGLE (System for Information on Grey Literature in Europe) | 1980 - 2005^*^ |
| Psycinfo | 1806-2012 |
| Web of Knowledge — ISI Proceedings | 1990 - 2010 |
| Web of Knowledge — ISI Science Citation Index | 1981 – 2012 |
| Web of Knowledge — ISI Social Science Citation Index | 1981 - 2012 |

We will not impose a language or publication restriction. We will first search MEDLINE (See appendix 1). From this, we will adapt subsequent search strategies for use in each database

**Searching other resources**

We will identify further studies for possible inclusion in our review by following the reference lists from studies located by our initial search strategies. A citation search will also be performed using the ‘cited by’ option on Scopus, and the ‘related articles’ option on PubMed and Web of Science. We will contact experts in the field for further unpublished or ongoing trials.

## 5.1 Data extraction and analysis

An independent double data extraction will be performed, using a pre-designed form adapted from...

The data extraction will be performed by MAD and LC on a summer internship at the University of Hertfordshire.

Inconsistencies will be resolved by discussion, moderated by BM. In the first stage of the review, we will assess the titles and abstracts of all articles retrieved by electronic searches. A full-text analysis of all citations meeting the selection criteria will then be conducted.

We will extract information about 1) the author(s), 2) publication year, 3) country, 4) type of study design, 5) aim(s) and research questions, 6) type of participants and sample size, 7) data collection method; 8) response rate, 9) method(s) of analysis, 10) outcomes.

Additional information about the duration of follow-up, characteristics of the intervention and control groups, as well as key findings, will be extracted from the interventional studies.

A narrative review will be produced independently of the heterogeneity of included studies.

In parallel, heterogeneity will be assessed using the Chi-Square test and I^2^ test (Higgins 2002). If there is sufficient homogeneity, studies will be pooled in a meta-analysis with dichotomous outcomes presented as relative risks (RR) and continuous data as mean differences (MD). However, in the likely event of heterogeneity studies will not be pooled but instead we will present data narratively with an indication of whether the effect of the intervention was positive, negative or not statistically significant. Where possible we will report dichotomous outcomes as relative risks and continuous data as mean differences, both with 95% confidence intervals.

5.3 Study quality assessment

In addition to the above selection criteria, the quality of all included studies will be considered and appraised. The quality assessment will be informed by the EPOC risk of bias tool (EPOC 2008), the PRISMA statements of standards for assessing systematic reviews (Moher et al. 2009), as well as the Jadad and CONSORT scales for RCTs (Jadad et al. 1996; Moher et al. 2001). The quality of qualitative studies will be assessed using Spencer’s framework (Spencer et al. 2003). The resulting checklist will be used by two independent assessors against all included studies. Discrepancies will be resolved by discussion and consensus.

# References

[Bal BS](http://www.ncbi.nlm.nih.gov/pubmed?term=%22Bal%20BS%22%5BAuthor%5D), [Choma TJ](http://www.ncbi.nlm.nih.gov/pubmed?term=%22Choma%20TJ%22%5BAuthor%5D). What to Disclose? Revisiting Informed Consent. [Clin Orthop Relat Res.](http://www.ncbi.nlm.nih.gov/pubmed/22215479) 2012 Jan 4 Epub ahead of print.

[Barry MJ](http://www.ncbi.nlm.nih.gov/pubmed?term=%22Barry%20MJ%22%5BAuthor%5D), [Wescott PH](http://www.ncbi.nlm.nih.gov/pubmed?term=%22Wescott%20PH%22%5BAuthor%5D), [Reifler EJ](http://www.ncbi.nlm.nih.gov/pubmed?term=%22Reifler%20EJ%22%5BAuthor%5D), [Chang Y](http://www.ncbi.nlm.nih.gov/pubmed?term=%22Chang%20Y%22%5BAuthor%5D), [Moulton BW](http://www.ncbi.nlm.nih.gov/pubmed?term=%22Moulton%20BW%22%5BAuthor%5D). Reactions of potential jurors to a hypothetical malpractice suit: alleging failure to perform a prostate-specific antigen test. [J Law Med Ethics.](http://www.ncbi.nlm.nih.gov/pubmed?term=Barry%20MJ%20jurors) 2008 Summer; 36(2):396-402, 214.

Charles C, Gafni A, Whelan T. Shared decision-making in the medical encounter: what does it mean? (Or it takes at least two to tango). *Social Science & Medicine*, 1997; 44: 681–692.

Coulter A, Cleary PD. Patients’ Experiences with Hospital Care in Five Countries. Health Affairs. 244, 247-48 (2001).

Edwards A, Elwyn G. Inside the black box of shared decision-making: distinguishing between the process of involvement and who makes the decision. Health Expectations. 2006, 9(4), 307-320.

Feldman-Stewart D. et al., Practical Issues in Assisting Shared Decision-Making, 3 Health Expectations 46, 49 (2000).

Grol A et al. *Patients in Europe Evaluate General Practice Care: An International Comparison*, Brit. J.Gen. Prac. 882, 884-86 (2000).

King JS, Moulton B. Rethinking informed consent: the case for Shared Medical Decision-making. American Journal of Law and Medicine. 2006;32(4):429-501.

[Stacey D](http://www.ncbi.nlm.nih.gov/pubmed?term=%22Stacey%20D%22%5BAuthor%5D), [Bennett CL](http://www.ncbi.nlm.nih.gov/pubmed?term=%22Bennett%20CL%22%5BAuthor%5D), [Barry MJ](http://www.ncbi.nlm.nih.gov/pubmed?term=%22Barry%20MJ%22%5BAuthor%5D), [Col NF](http://www.ncbi.nlm.nih.gov/pubmed?term=%22Col%20NF%22%5BAuthor%5D), [Eden KB](http://www.ncbi.nlm.nih.gov/pubmed?term=%22Eden%20KB%22%5BAuthor%5D), [Holmes-Rovner M](http://www.ncbi.nlm.nih.gov/pubmed?term=%22Holmes-Rovner%20M%22%5BAuthor%5D), [Llewellyn-Thomas H](http://www.ncbi.nlm.nih.gov/pubmed?term=%22Llewellyn-Thomas%20H%22%5BAuthor%5D), [Lyddiatt A](http://www.ncbi.nlm.nih.gov/pubmed?term=%22Lyddiatt%20A%22%5BAuthor%5D), [Légaré F](http://www.ncbi.nlm.nih.gov/pubmed?term=%22L%C3%A9gar%C3%A9%20F%22%5BAuthor%5D), [Thomson R](http://www.ncbi.nlm.nih.gov/pubmed?term=%22Thomson%20R%22%5BAuthor%5D). Decision aids for people facing health treatment or screening decisions. [Cochrane Database Syst Rev.](http://www.ncbi.nlm.nih.gov/pubmed/21975733) 2011 Oct 5;(10):CD001.
