# Supplementary material for: Can shared decision-making reduce medical malpractice litigation? A systematic review
Source: BMC Health Serv Res. 2015 Apr 18;15:167. doi: 10.1186/s12913-015-0823-2 (PMC4409730; doi:10.1186/s12913-015-0823-2)
Supplement: Additional file 3: — Search strategy. [file 12913_2015_823_MOESM3_ESM.docx]

**Search Strategy**

**Shared decision making & litigation**

**Appendix 1**

**Search Strategy - OVID Medline 1946- June 2012**

1. exp Decision-making/

2. *"Patient Participation"/

3. Physician-Patient Relations/

4. doctor-patient relationship.tw.

5. (patient adj3 decision-making).tw.

6. (share* adj decision adj mak*).ti,ab.

7. (share* adj decision adj mak*).tw.

8. ((decis* or information) adj3 (choic* or aid*)).tw.

9. shared decision-making.mp.

10. informed decision*.tw.

11. (decision support adj (strat* or method*or technique* or intervention*)).tw.

12. *Negotiating/

13. (engage patients adj3 shared decision*).tw.

14. decision?making.mp.

15. shared decision*.tw.

16. shared decision-making.mp.

17. decisionmaking.tw.

18. making decision*.mp.

19. patient preference*.mp.

20. or/1-19

21. exp Decision Support Techniques/

22. exp Decision Support Systems, Clinical/

23. decision-making technique*.tw.

24. decision support method*.tw.

25. (decision adj3 (aids or strat* or method*or technique* or intervention*)).tw.

26. (decision support adj (strat* or method*or technique* or intervention*)).tw.

27. (decision-making adj3 (programme* or approach*)).tw.

28. or/21-27

29. 20 or 28

30. litigat*.tw.

31. medical malpractice cases.mp.

32. medical malpractice.tw.

33. legal liabilit*.tw.

34. medical negligence claim*.mp.

35. court case.tw.

36. legal proceeding*.tw.

37. (lawsuit or lawsuites).mp.

38. legal action.tw.

39. liabilit* risk.mp.

40. *"Malpractice"/

41. Disclosure/lj [Legislation & Jurisprudence]

42. Legal Cases/

43. *"Liability, Legal"/

44. or/30-43

45. *Communication/

46. *"Access to Information"/

47. (informed adj2 (consent or decision* or choice*)).tw.

48. Informed Consent/

49. Health Communication/

50. communicat*.tw.

51. lack of communication.mp.

52. Education, Medical, Graduate/ or Clinical Competence/ or Education, Medical, Continuing/ or Education, Medical/ or "Attitude of Health Personnel"/

53. or/45-52

54. 20 or 53

55. (Impact or Effective* or Efficien* or Useful* or Valuable).mp.

56. 29 and 44

57. 44 and 53 and 55

58. 56 or 5
